# Turkish UCLA PTSD‐RI‐5: Validation and the Role of Symptom Severity in Functional Impairment

**DOI:** 10.1002/mpr.70101

**Published:** 2026-08-06

**Authors:** Melek Ince, Fatma Sibel Durak, Bugra Ince, Alan Steinberg

**Affiliations:** ^1^ Department of Child and Adolescent Psychiatry Izmir Faculty of Medicine, University of Health Sciences Dr. Behçet Uz Children's Hospital Izmir Türkiye; ^2^ Department of Physical and Rehabilitation Medicine Izmir Faculty of Medicine, University of Health Sciences Izmir City Hospital Izmir Türkiye; ^3^ Department of Psychiatry and Biobehavioral Sciences UCLA/Duke University National Center for Child Traumatic Stress University of California Los Angeles California USA

**Keywords:** adolescents, functional impairment, PTSD, Reaction Index, Turkish

## Abstract

**Objectives:**

This study translated the UCLA PTSD Reaction Index for DSM‐5 into Turkish (PTSD‐RI‐5‐TR), evaluated its psychometric properties, and examined whether PTSD symptom severity predicts functional impairment in Turkish adolescents aged 11–17.

**Methods:**

A total of 157 trauma‐exposed adolescents from a tertiary Child and Adolescent Psychiatry Clinic participated. Internal consistency and test–retest reliability were evaluated. Construct validity was examined via confirmatory factor analysis (CFA) of a five‐factor model. Convergent, divergent, criterion, and known‐groups validity were tested. ROC analysis assessed diagnostic accuracy. Binary logistic regression was used to evaluate the impact of scores on four functional domains.

**Results:**

PTSD‐RI‐5‐TR showed good internal consistency and test–retest reliability (acceptable for Category C). CFA indicated excellent fit (CMIN/DF, CFI, TLI, RMSEA) and good fit (GFI, NFI). Scores correlated strongly with NSESSS‐PTSD and moderately with DES‐B. PTSD diagnoses were associated with significantly higher scores. ROC analysis yielded an AUC = 0.909, sensitivity = 86.4%, specificity = 84.1% with an optimal cutoff score of 49.5. In total score models, each one‐point increase predicted impairment in developmental progression (OR = 1.097), home (OR = 1.084), peer (OR = 1.071), and school functioning (OR = 1.063). In symptom cluster models, negative mood/cognition (D) most strongly predicted peer (OR = 1.205) and developmental impairment (OR = 1.155); avoidance (C) predicted home impairment (OR = 1.250); arousal/reactivity (E) predicted home (OR = 1.144) and school impairment (OR = 1.131); intrusion (B) showed no independent effect.

**Conclusion:**

PTSD‐RI‐5‐TR is a reliable, valid tool for assessing PTSD in Turkish adolescents. Higher total PTSD scores and higher scores in the C, D, and E symptom clusters may indicate a greater likelihood of functional impairment.

**Trial Registration:**

This study was prospectively registered at ClinicalTrials.gov (Identifier: NCT06077474 {2023‐10‐05})

## 1 Introduction

Exposure to potentially traumatic events is highly prevalent during childhood and adolescence, with recent epidemiological studies indicating that approximately one‐third (31.1%) to more than half (58.4%) of young people experience at least one traumatic event during their lifetime (Carliner et al. 2017; Lewis et al. 2019). While many trauma‐exposed youth do not develop clinical post‐traumatic stress disorder (PTSD), a significant subgroup remains at high risk, with estimated prevalence rates ranging between 12.0% and 20.3% depending on the diagnostic criteria applied (Visser et al. 2026). PTSD symptoms in youth include those among the DSM‐5 B, C, D, and E subcategory symptoms of *Intrusion*, *Avoidance*, *Negative Cognition*, and *Arousal/Reactivity* (APA 2013). Early identification of these symptoms is essential for timely intervention and the prevention of long‐term psychological impairment, functional and behavioral disturbances, and developmental disruptions.

A variety of methods are used to assess PTSD symptoms in children/adolescents, including clinical self‐report interviews with the traumatized child/adolescent, parent/caregiver interviews, and self‐administered measures. Among these, clinical self‐report interviews with youth are particularly valuable for detecting internalizing symptoms that may not be recognized by parents/caregivers.

The UCLA PTSD Reaction Index (PTSD‐RI) is the most widely used PTSD assessment in clinical and research settings, has been translated into numerous languages, and its psychometric properties have been well documented (Steinberg et al. 2004). Following the DSM‐5 revision of PTSD in 2013 which added three new symptoms (negative expectations about the self, others, or the world; distorted blame; and reckless or self‐destructive behavior) and reorganized the disorder into four categories (APA 2013), the Reaction Index was updated to the 31‐item PTSD‐RI‐5. The revised scale now assesses Criterion A exposure plus the four DSM‐5 B, C, D, and E categories The PTSD‐RI‐5 includes two complementary versions: a self‐report form for children and adolescents, and a caregiver‐report form that provides for a comprehensive assessment from both the child's and the caregiver's perspectives, further contributing to its widespread use in the evaluation of trauma‐related symptoms (Kaplow et al. 2020; Ramos et al. 2021; Steinberg et al. 2013).

Initial studies in clinical samples and in multi‐site Japanese cohorts have confirmed its solid internal consistency, criterion referenced validity, and diagnostic accuracy (Takada et al. 2018). A cross‐national study conducted in 11 countries also confirmed the scale's four‐factor structure and strong internal reliability (*α* = 0.90–0.94), though some variability in factor loadings across cultures was observed (Doric et al. 2019).

Previous research has shown that PTSD symptom severity in children and adolescents is not only associated with psychological distress but also with significant impairment in daily functioning across home, school, and social domains (Bartels et al. 2024; Carrion et al. 2002; Steinberg et al. 2013, 2019). Among symptom clusters, negative mood/cognition (D) and arousal/reactivity (E) have been identified as the strongest predictors of functional impairment (Bartels et al. 2024). These findings highlight that PTSD in youth may disrupt developmental processes and daily functioning, underscoring the importance of evaluating not only symptom presence but also its functional consequences.

While the present study was underway, a Turkish adaptation of the PTSD‐RI‐5 was published (Kucukardali et al. 2025). Although their study provided important evidence for the psychometric validity of the Turkish PTSD‐RI‐5, their criterion‐related analyses relied on general anxiety and depression measures and did not include a dissociation‐specific instrument. The present study extends this work by testing a five‐factor model that includes dissociative symptoms and by examining disorder‐specific criterion validity. In addition, it evaluates the relationship between PTSD symptom severity, specific symptom clusters, and functional impairment across home, school, peer, and developmental domains. We hypothesized that (1) the PTSD‐RI‐5‐TR would demonstrate adequate validity and reliability in Turkish adolescents aged 11–17 years and (2) PTSD symptom severity would significantly predict functional impairment.

## 2 Method

### 2.1 Participants

The study sample consisted of 157 adolescents aged 11–17 years who had experienced at least one traumatic event, as determined through clinical interview. Most participants had experienced interpersonal trauma, with extrafamilial sexual abuse being the most frequently reported trauma type. The sample size was calculated to include a minimum of five participants per item of the scale (Campo‐Arias and Oviedo 2008). Exclusion criteria included psychiatric disorders such as schizophrenia, bipolar disorder, and autism spectrum disorder as well as significant cognitive or neurological impairments that could interfere with the assessment of psychiatric symptoms. All participants were recruited from the Child and Adolescent Psychiatry Clinic at Dr. Behçet Uz Children's Hospital, University of Health Sciences, Izmir, Türkiye. Data was collected between October 2023 and August 2024. The study was approved by the local institutional ethics committee (Decision No. 2023/12‐06) and conducted in accordance with the Declaration of Helsinki. Written informed consent was obtained from all participants and their legal guardians. This study was prospectively registered at ClinicalTrials.gov (Identifier: NCT06077474 {2023‐10‐05}).

### 2.2 Translation and Adaptation

Adaptation followed a standard forward‐backward translation protocol (Beaton et al. 2000): two native‐Turkish bilinguals produced independent forward translations, which were reconciled into a single draft; two native‐English bilinguals, blind to the original, back‐translated this draft; all versions were compared by the translators and study team to resolve discrepancies and a preliminary version of the scale was developed. This version was tested on 10 patients not included in the validity and reliability study (cognitive debriefing) to identify potential issues related to wording, terminology, clarity, and comprehension. Based on the feedback, final modifications were made, and the scale was finalized.

### 2.3 Measures

#### 2.3.1 UCLA PTSD Reaction Index for DSM‐5 (PTSD‐RI‐5)

The PTSD‐RI‐5 was originally developed as a self‐report scale that screens for trauma exposure and assesses DSM‐5 PTSD symptoms in children and adolescents (Pynoos et al. 1993; Steinberg et al. 2004). It includes: (1) a comprehensive trauma history section that allows for identification of exposure to 19 different types of traumas, trauma‐specific features of that exposure, type of exposure (victim, witnessed, learned about), and age(s) of exposure; (2) a section that includes 27 core PTSD symptoms; and (3) a section examining clinically significant distress and disturbances in behavior and functioning at home, in school, with peers, and in development progression. Four items assess reactions that contribute to meeting criteria for Dissociative Subtype. Frequency of symptoms experienced over the past month are rated on a five‐point scale (0 = never to 4 = most of the time). Total scores range from 0 to 80 and map onto the four DSM‐5 symptom categories. Scores of 35 and above indicate likelihood of meeting full diagnostic criteria for PTSD, with higher scores reflecting greater severity (Kaplow et al. 2020).

#### 2.3.2 DSM‐5 Posttraumatic Stress Symptom Severity Scale–Child Form—National Stressful Events Survey PTSD Short Scale (NSESSS‐PTSD)

NSESSS‐PTSD is a nine‐item measure that assesses the severity of posttraumatic stress disorder in children ages 11–17 following an extremely stressful event. The measure was designed to be completed by the child upon receiving a diagnosis of posttraumatic stress disorder (or clinically significant posttraumatic stress disorder symptoms) and thereafter completed in follow‐up visits with the clinician. Each item asks the child receiving care to rate the severity of his or her post‐traumatic stress symptoms. Each item is rated on a five‐point scale from 0 (“not at all”) to 4 (“extremely”), yielding a total raw score between 0 and 36; higher scores denote greater symptom severity. The nine items are summed to obtain the raw total and then divided by nine to generate an average total score, which places severity on a 0–4 continuum: none, mild, moderate, severe, or extreme. The Turkish version has demonstrated robust reliability and validity in a study conducted by Sapmaz et al. (Ş. Yalin Sapmaz et al. 2016).

#### 2.3.3 DSM‐5 Dissociative Symptoms Severity Scale‐Child Form‐Brief Dissociative Experiences Scale–Modified (DES‐B)

The Brief Dissociative Experiences Scale (DES‐B)—Modified is an 8‐item measure that assesses the severity of dissociative experiences in children ages 11–17. The measure is completed by the child upon receiving a diagnosis of a Dissociative Disorder (or clinically significant dissociative symptoms) and thereafter in follow‐up visits with the clinician. Each item asks the child receiving care to rate the severity of his or her dissociative experiences during the past 7 days. Each item is rated on a five‐point scale (0 = not at all to 4 = more than once a day), yielding a total raw score of 0–32; higher scores reflect greater dissociative symptom severity. The Turkish version has demonstrated robust reliability and validity in the study conducted by Yalin Sapmaz et al. (2017).

PTSD diagnosis was established through a clinical interview based on PTSD sections of the Kiddie Schedule for Affective Disorders and Schizophrenia present version for DSM‐5 (K‐SADS). The PTSD RI‐5 was administered by the clinician to the children/adolescents who agreed to participate. The Trauma History and Trauma Details sections were administered by the clinician. The PTSD Symptom Scale was completed by children who were deemed to have the ability to complete it. In line with the questionnaire procedure, participants reported all traumatic events they had experienced and identified the event that bothered them the most. Subsequent analyses were conducted using this primary trauma type. The Distress and Impairment in Functioning section was completed by the clinician at the end of the interview. Sociodemographic data, the DSM‐5 Posttraumatic Stress Symptom Severity Scale—Child Age 11–17 (NSESSS‐PTSD), and the DSM‐5 Dissociative Symptoms Severity Scale—Child Form (DES‐B) were collected from each participant. Test–retest reliability of the PTSD‐RI‐5‐TR was evaluated in a subsample of 30 adolescents who repeated the scale 2 weeks later.

### 2.4 Statistical Analysis

All statistical analyses were performed using IBM SPSS Statistics version 26.0 (IBM Corp., Armonk, NY, USA) and IBM SPSS AMOS version 24.0. Group comparisons were made using chi‐square or Fisher's exact tests for categorical variables, and independent‐samples *t*‐test or Mann–Whitney *U* test for continuous variables. Reliability was evaluated using Cronbach's alpha and the Intraclass Correlation Coefficient (ICC). Alpha values between 0.70 and 0.95 were considered acceptable (Tavakol and Dennick 2011). ICCs were interpreted as follows: < 0.40 = poor, 0.40–0.59 = moderate, 0.60–0.74 = good, and ≥ 0.75 = excellent reliability (Koo and Li 2016). Construct validity was assessed by Confirmatory Factor Analysis (CFA) based on a five‐factor model. Model fit was evaluated using the Chi‐Square/Degrees of Freedom ratio (CMIN/DF), Comparative Fit Index (CFI), Tucker–Lewis Index (TLI), Normed Fit Index (NFI), Goodness‐of‐Fit Index (GFI), and Root Mean Square Error of Approximation (RMSEA). Acceptable thresholds were as follows: CMIN/DF < 5 (good) and < 2 (excellent) (Whittaker and Schumacker 2022), CFI, TLI, NFI, and GFI > 0.90 (good) and > 0.95 (excellent) (Baumgartner and Homburg 1996). RMSEA < 0.08 (good) and < 0.05 (excellent) (Hu and Bentler 2009). Convergent and divergent validity were examined using Spearman's correlation coefficients between items and their respective subscales. Convergent validity was considered acceptable when each item showed a correlation (*ρ*) > 0.40 with its corresponding symptom category. Divergent validity was supported if the correlation of an item with its own symptom category was higher than its correlations with other symptom categories. Criterion‐referenced validity was assessed through Spearman correlations between the total PTSD‐RI‐5‐TR score, and the NSESSS‐PTSD and DES‐B. Correlation coefficients were interpreted as ≥ 0.70 strong, 0.50–0.69 moderate, and 0.30–0.49 low (Bland and Altman 1994; Mukaka 2012; Schober and Schwarte 2018). Known‐groups validity was evaluated by comparing participants with and without a clinical PTSD diagnosis. Diagnostic performance was analyzed via Receiver Operating Characteristic (ROC) analysis. The discriminative capacity of the PTSD‐RI‐5‐TR was interpreted according to the Area Under the Curve (AUC) values: > 0.50 represented discriminative capacity, 0.70–0.80 indicated moderate performance, 0.80–0.90 good performance, and 0.90–1.00 excellent performance (Ekelund 2012). To examine the impact of posttraumatic stress symptom severity and symptom profiles on different domains of functional impairment, a series of binary logistic regression analyses were performed. A score of '0' (None), indicating no interference in the respective domain, was coded as 'No Impairment' (0). Any score of '1' or higher ('Little' to 'Most') was categorized as 'Impairment' (1), reflecting the threshold at which symptoms begin to cause clinically significant distress or functional interference as per DSM‐5 Criterion G. All analyses were conducted using a significance level of *p* < 0.05.

## 3 Results

For the total of 157 patients who participated in the study, 88 were diagnosed with PTSD, while 69 did not meet PTSD criteria. The majority (*n* = 132, 84%) were female. The most frequently reported type of trauma was extrafamilial sexual abuse (*n* = 107, 68%). 34.8% of participants reported exposure to more than one trauma type. In most cases (*n* = 138, 88%), the adolescents had been victims of the traumatic event. Significant group differences emerged only with respect to educational level and self‐harm behaviors. Middle school students showed a significantly lower rate of PTSD (*p* = 0.006), whereas self‐harm was significantly more common among patients with PTSD (*p* = 0.001). The demographic data and group comparisons are presented in Table 1.

**TABLE 1:** Demographic and clinical characteristics of participants.

	PTSD (*N* = 88)	Non‐ PTSD (*N* = 69)	*p* value
Gender, *N* (%)			
Female	76 (86.4)	56 (81.2)	0.376 (*χ* ^2^)
Male	12 (13.6)	13 (18.8)	
Age, median (IQR)	15 (2)	14 (3.5)	0.061 (m)
Age at the time of the trauma, median (IQR)	13 (4)	12 (3.5)	0.174 (m)
Trauma type, *N* (%)			
Sexual assault	61 (69.3)	46 (66.7)	0.073 (e)
Sexual abuse	6 (6.8)	2 (2.9)	
Serious accidental injury	5 (5.7)	1 (1.1)	
Illness	1 (1.1)	0 (0)	
Community violence	1 (1.1)	1 (1.1)	
Domestic violence	1 (1.1)	2 (2.9)	
Physical assault	1 (1.1)	0 (0)	
Physical abuse	0 (0)	3 (4.3)	
Disaster	1 (1.1)	4 (5.8)	
Neglect	1 (1.1)	0 (0)	
Psychological maltreatment	1 (1.1)	1 (1.1)	
Interference with caregiving	1 (1.1)	0 (0)	
Kidnapping	1 (1.1)	0 (0)	
Terrorism	1 (1.1)	0 (0)	
Bereavement	0 (0)	5 (7.2)	
Separation	3 (3.4)	2 (2.9)	
Bullying	1 (1.1)	2 (2.9)	
Witnessed suicide	1 (1.1)	0 (0)	
School violence	1 (1.1)	0 (0)	
Role in event, *N* (%)			
Victim	77 (87.5)	61 (88.4)	0.863 (*χ* ^2^)
Witness	11 (12.5)	8 (11.6)	
Level of education, *N* (%)			
School dropout	10 (11.4)	4 (5.8)	0.034 (e)
Middle school	18 (20.5)^†^	28 (40.6)^†^	
High school	58 (65.9)	36 (52.2)	
Open (Distance) high school	2 (2.3)	1 (1.4)	
Self‐injurious behavior, *N* (%)	14 (15.9)	0 (0)	0.001 (*χ* ^2^)
Psychiatric admission history, *N* (%)			
Present	30 (34.1)	33 (47.8)	0.081 (*χ* ^2^)
Absent	58 (65.9)	36 (52.2)	
History of psychiatric hospitalization, *N* (%)			
Present	3 (3.4)	2 (2.9)	1.0 (e)
Absent	85 (96.6)	67 (97.1)	
History of substance abuse, *N* (%)			
Present	4 (4.5)	0 (0)	0.131 (e)
Absent	84 (95.5)	69 (100)	
Loss of a parent, *N* (%)			
Yes	9 (10.7)	8 (12.3)	0.762 (*χ* ^2^)
No	75 (89.3)	57 (87.7)	
Smoking or alcohol use, *N* (%)			
Yes	7 (8.0)	4 (5.8)	0.756 (e)
No	81 (92.0)	65 (94.2)	

Abbreviations: e, Exact test; m, Mann Whitney U test; *χ* ^*2*^, Chi‐square test.

^†^ bonferroni‐corrected post‐hoc z‐test indicated a significant between‐group difference for the middle school category (adjusted *p* = 0.006).

Major depressive disorder and anxiety disorders were significantly more common in the PTSD group, whereas other psychiatric comorbidities and psychopharmacological treatment rates did not differ significantly between the groups (Table 2).

**TABLE 2:** Comorbidities and medications in the study sample.

*N* (%)	PTSD (*N* = 88)	Non‐ PTSD (*N* = 69)	*p* value
Comorbidities			< 0.001
Major depressive disorder^a^	37 (42.0)	3 (4.3)	
Attention‐deficit hyperactivity disorder	3 (3.4)	6 (8.7)	
Oppositional Defiant and conduct disorders	9 (10.2)	2 (2.9)	
Communication disorders	2 (2.3)	0 (0)	
Dissociative identity disorders	1 (1.1)	1 (1.4)	
Anxiety Disorders^a^	6 (6.8)	0 (0)	
Substance use disorders	1 (1.1)	0 (0)	
Medications			
Antidepressants	31 (30.4)	18 (24.0)	0.732
Antipsychotics	18 (17.6)	11 (14.7)	
Stimulant and Nonstimulant agents	5 (4.9)	6 (8.0)	
Benzodiazepines	2 (2.0)	1 (1.3)	

^a^ Indicates statistically significant between‐group differences in post‐hoc analyses.

Reliability analyses indicated that internal consistency levels for the PTSD‐RI‐5‐TR were optimal across all categories, with Cronbach's *α* values ranging from 0.774 to 0.931, except for Category C (*α* = 0.651), which was still considered acceptable based on other metrics (e.g., an item–total correlation of *r* = 0.484). ICCs demonstrated excellent test–retest reliability for Category E (ICC = 0.772), good reliability for Categories C (ICC = 0.613), D (ICC = 0.740), and the total score (ICC = 0.707), and moderate reliability for Category B (ICC = 0.483) and dissociative symptoms (ICC = 0.436). Among the items in Category B, items B1, B4, and B5 showed poor ICC values (range = 0.127–0.360), B2 showed moderate reliability (ICC = 0.557), and B3 good reliability (ICC = 0.621). For the dissociative subtype, item A1 demonstrated moderate reliability (ICC = 0.443) and item A2 poor reliability (ICC = 0.293).

**FIGURE 1:**
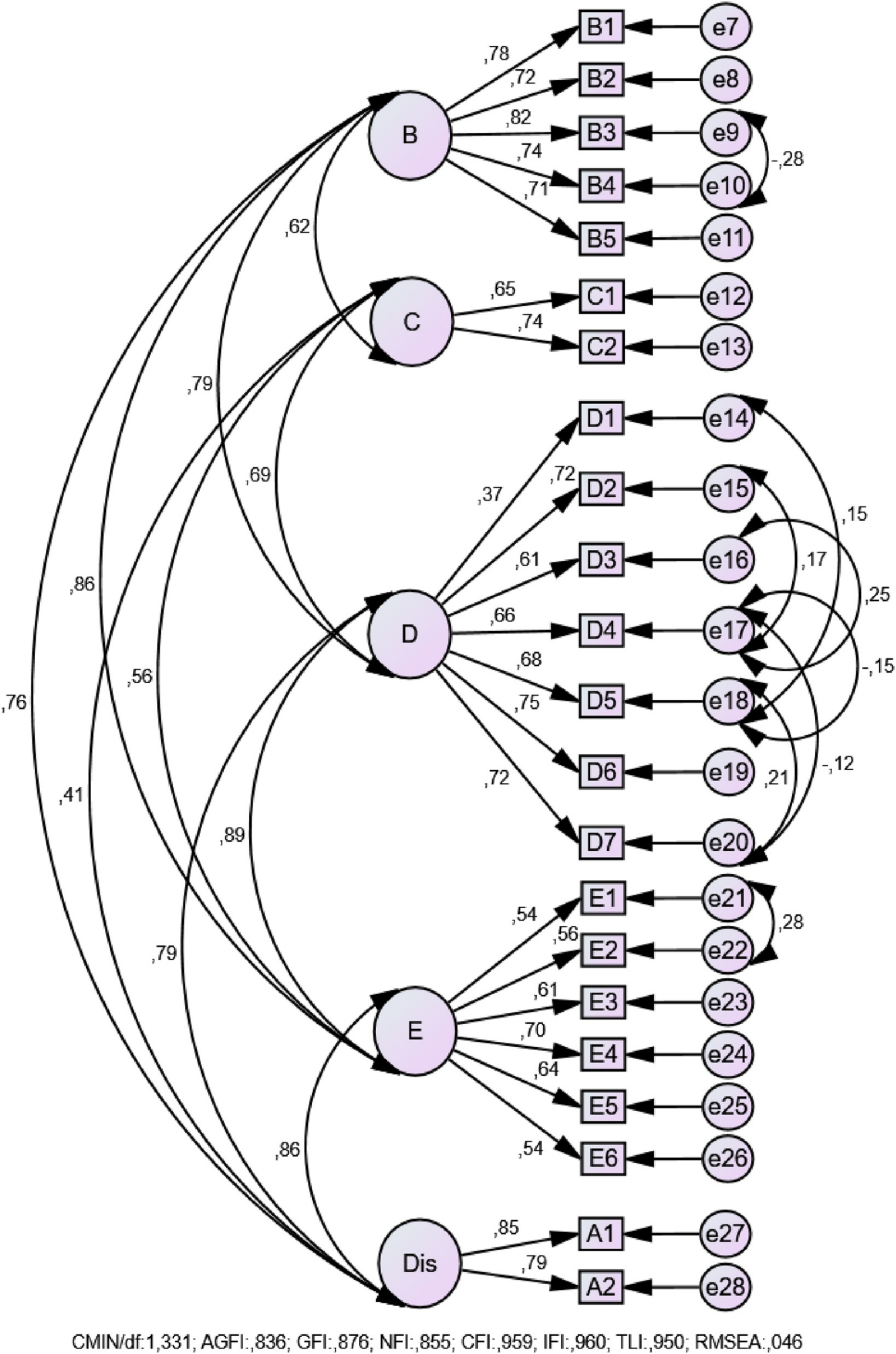
Factor structure of PTSD‐RI‐5‐Turkish.

Confirmatory factor analysis (CFA) results demonstrated excellent model fit based on indices such as CMIN/DF, CFI, TLI, and RMSEA. Although the GFI (0.876) and NFI (0.855) values were slightly below the established thresholds for good fit, they remained close to acceptable levels (see Figure 1).

All items demonstrated adequate convergent validity (Spearman's rho = 0.534–0.894) and clear divergent validity. Regarding criterion‐referenced validity, a statistically significant strong correlation was found between the PTSD‐RI‐5‐TR total score and the NSESSS‐PTSD (*ρ* = 0.750, *p* < 0.001), and a moderate correlation between the dissociative subscale and the DES‐B (*ρ* = 0.547, *p* < 0.001).

Regarding known‐groups validity, participants diagnosed with PTSD had significantly higher PTSD‐RI‐5‐TR total scores compared to those without a PTSD diagnosis (mean difference = 25.2; 95% CI: 20.8–29.6), *t* (115.951) = 11.393, *p* < 0.001 (Table 3).

**TABLE 3:** PTSD‐RI‐5‐TR category scores among participants with and without a PTSD diagnosis.

Score	PTSD (*N* = 88)	Non‐PTSD (*N* = 69)	*p* value
B category, median (IQR)	15 (6)	7 (8)	< 0.001^m^
C category, median (IQR)	6 (2)	4 (4)	< 0.001^m^
D category, median (IQR)	22 (6)	14 (9.5)	< 0.001^m^
E category, median (IQR)	18 (6)	9 (9)	< 0.001^m^
Dissociative category, median (IQR)	6 (4)	2 (5)	< 0.001^m^
Total score, mean (SD)	59.1 (10.8)	33.9 (15.6)	< 0.001^t^

Abbreviations: IQR, interquartile range; SD, standard deviation.

^m^ Mann Whitney *U* test.

^t^ Independent sample *t* test.

**FIGURE 2:**
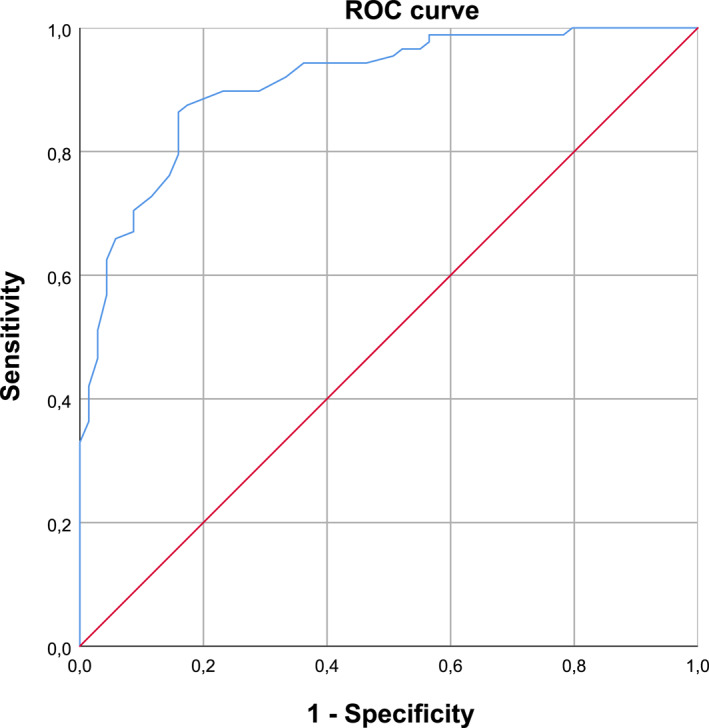
The discriminative capacity of the Turkish PTSD‐RI‐5. AUC = 0.909 (95% CI = 0.864–0.954), *p* < 0.001.

In the ROC analysis, the total score of the PTSD‐RI‐5‐TR achieved the maximum Youden index at a cutoff value of 49.5, yielding a sensitivity of 0.864 and a specificity of 0.841. The area under the curve (AUC) was 0.909 (95% CI = 0.864–0.954), *p* < 0.001 (Figure 2).

Based on the categorical criteria of the PTSD‐RI‐5‐TR, the classification accuracy for identifying PTSD demonstrated a sensitivity of 80.6% and a specificity of 78.2%. The positive predictive value (PPV) was 82.5%, and the negative predictive value (NPV) was 76.0%.

Logistic regression analyses were conducted to examine the predictive role of PTSD symptom severity (total score) and symptom profiles (intrusion, avoidance, negative mood, and arousal) on various domains of functional impairment (at home, in school, in peer relations, and in developmental progression) (Table 4).

**TABLE 4:** Functional impairment models based on PTSD score and symptom profile.

		Variables	B	S. E	*p* value	Odds ratio	95% CI for odds ratio
Model 1 (PTSD score)							
	Home	PTSD score	0.081	0.014	0.000	1.084	1.056–1.114
	School	PTSD score	0.062	0.012	0.000	1.063	1.040–1.088
	Peer relationships	PTSD score	0.068	0.013	0.000	1.071	1.045–1.097
	Developmental progression	PTSD score	0.092	0.015	0.000	1.097	1.065–1.129
Model 2 (symptom profile)							
	Home	B score	0.036	0.051	0.477	1.037	0.938–1.145
		C score	0.223	0.098	0.022	1.250	1.032–1.513
		D score	0.044	0.045	0.330	1.045	0.957–1.141
		E score	0.135	0.052	0.009	1.144	1.034–1.266
	School	B score	−0.042	0.049	0.388	0.959	0.870–1.055
		C score	0.154	0.090	0.087	1.167	0.978–1.392
		D score	0.076	0.043	0.073	1.079	0.993–1.174
		E score	0.123	0.048	0.009	1.131	1.031–1.242
	Peer relationships	B score	0.075	0.049	0.122	1.078	0.980–1.186
		C score	−0.029	0.095	0.761	0.972	0.807–1.170
		D score	0.187	0.047	0.000	1.205	1.099–1.322
		E score	−0.038	0.049	0.431	0.962	0.874–1.059
	Developmental progression	B score	0.059	0.051	0.242	1.061	0.961–1.172
		C score	0.079	0.100	0.428	1.082	0.890–1.316
		D score	0.144	0.047	0.002	1.155	1.054–1.267
		E score	0.070	0.050	0.164	1.072	0.972–1.184

All models based on total PTSD score (Model 1) were statistically significant (*p* < 0.001), indicating that higher PTSD severity was associated with an increased likelihood of functional impairment across all domains. Specifically, each unit increase in PTSD total score was associated with a higher odd of impairment at home (OR = 1.084), at school (OR = 1.063), in peer relationships (OR = 1.071), and in developmental progression (OR = 1.097), all with narrow and significant confidence intervals. Symptom profile–based models (Model 2) were also statistically significant overall and provided additional insight into which symptom categories contributed most strongly to functional impairment. Avoidance (C) and Arousal (E) symptoms significantly predicted home‐related impairment (OR = 1.250 and 1.144, respectively; *p* < 0.05). Arousal symptoms were also a significant predictor of school‐related impairment (OR = 1.131, *p* = 0.009). Peer‐related impairment was significantly associated with Negative Mood Symptoms (D) (OR = 1.205, *p* < 0.001), while Developmental Impairment was predicted by the same category (OR = 1.155, *p* = 0.002).

## 4 Discussion

In this study, the PTSD‐RI‐5 was adapted into Turkish and subsequently analyzed for validity and reliability. Our findings indicate that the scale, when adapted for adolescents aged 11–17, preserves its psychometric properties in the Turkish version. Our reliability analyses indicated that the internal consistency of the PTSD‐RI‐5‐TR was optimal across all categories except for Category C. This finding aligns with previous studies conducted by the scale's developers and other psychometric evaluations, including the recent Turkish validation by Kaplow et al. (2020); Kucukardali et al. (2025); Steinberg et al. (2013). However, unlike Kucukardali et al. (2025) dissociative symptom items demonstrated acceptable internal consistency in our sample. In a Japanese study examining reliability and validity, although Category C demonstrated an optimal Cronbach's alpha of 0.85 for the overall sample, it exhibited the lowest reliability when the sample was divided into different age subgroups (Takada et al. 2018). It is widely recognized that Cronbach's alpha is sensitive to the number of items on a scale. Hence, the lower alpha for Category C, which comprises only two questions, could be attributed to this factor. In our study, based on the other metrics assessed (e.g., item–total correlations), Category C was found to be acceptable (*r* = 0.484). This outcome is in line with the developers' own findings (Kaplow et al. 2020). However, in a multi‐center reliability and validity study spanning 11 countries, a Spearman–Brown analysis‐which is more resilient to having fewer items still produced coefficients ranging from 0.38 to 0.69 (Doric et al. 2019). This suggests the possibility of heterogeneous covariances among items in Category C, raising doubts about the subscale's unidimensionality. Although the inconsistencies in the literature imply that Category C of the scale may exhibit a lower reliability profile, this finding should be interpreted with consideration of possible cross‐cultural influences. Variations in how items are perceived across cultures could plausibly account for the observed differences in reliability.

Küçükardalı et al. reported excellent test–retest reliability at the total score level (ICC = 0.95) (Kucukardali et al. 2025). In contrast, our study evaluated test–retest reliability at both total‐subscale and item levels, providing a more detailed perspective. Category E showed excellent test–retest reliability, whereas Categories C, D, and the total score exhibited good reliability. In Category B, specifically items B1, B4, and B5 had weak ICCs, possibly reflecting a higher dependence on individual triggers. When conducting test–retest reliability studies, a 2‐week interval is commonly recommended to minimize recall bias and ensure that the participant's clinical status remains stable during the retest period (Koo and Li 2016). Although this timeframe is generally considered short enough to prevent significant changes in medical or psychological status, some items‐particularly those in Category B may still be sensitive to fluctuations that occur within this period. Exposure to or avoidance of specific trauma‐related triggers during these 2 weeks could not be controlled and may have influenced participants' responses. Therefore, the weaker ICC values may reflect the nature of these phasic symptoms (potentially influenced by exposure to trauma reminders or nightmares occurring during the interval) rather than a flaw in the scale's overall reliability. Although these findings do not undermine the overall reliability of the PTSD‐RI‐5‐TR, short‐term changes in items B1, B4, B5, and A2 should be interpreted cautiously. In clinical follow‐up, clinicians should avoid basing treatment decisions on isolated item changes and should instead consider total and subscale scores, recent exposure to trauma reminders, clinical interview findings, and, when available, information from caregivers. Repeated assessments under similar clinical conditions may also help distinguish transient symptom fluctuations from meaningful clinical change.

Previous research on confirmatory factor analyses has generally focused on four‐factor models of the PTSD‐RI‐5, aligned with DSM‐5's four core symptom categories (Kaplow et al. 2020; Takada et al. 2018). This has been justified by treating dissociative symptoms as a comorbid diagnostic feature. However, we argue that this approach does not fully reflect the scale's structural integrity. Although it is true that dissociative symptoms constitute a distinct diagnostic feature, these four items are administered as the continuation of the scale; consequently, the instrument should be assessed comprehensively. The design of the scale in accordance with DSM‐5 criteria does not, in our view, provide grounds for excluding a potential five‐factor structure as the goal is to test the scale's validity, not merely the DSM‐5 criteria. Notably, our study is the first to propose and successfully demonstrate a five‐factor model encompassing all items. While most international validations have shown good fit, a few countries have reported lower model fit (Takada et al. 2018). Our findings suggest that the PTSD‐RI‐5‐TR can be comprehensively and validly evaluated under a five‐factor model that incorporates dissociative items. Accordingly, the construct validity of the PTSD‐RI‐5‐TR was largely preserved due to its good model fit, along with convergent and divergent validity across all items.

Our study also demonstrated strong criterion referenced validity for the PTSD‐RI‐5‐TR. This result aligns with findings reported by the scale's developers (Kaplow et al. 2020) and in other international validation studies (Doric et al. 2019; Takada et al. 2018). Similarly, Küçükardalı et al. supported the validity of the PTSD‐RI‐5 through its correlations with measures of anxiety and depression (RCADS) (Kucukardali et al. 2025). In contrast, our study established criterion validity based on correlations with PTSD‐specific symptom severity (NSESSS‐PTSD), providing more direct evidence for disorder‐specific construct validity. Additionally, we are the first to specifically assess the criterion validity of the dissociative subscale, detecting a moderate correlation. While this is an encouraging finding, no comparable data exists in the literature. Future research on the psychometric performance of the dissociative subtype scale is warranted.

In both Turkish validation studies—Kucukardali et al. (2025) and the present one‐individuals diagnosed with PTSD had significantly higher scale scores (*p* < 0.001). This finding supports the ability of the PTSD‐RI‐5‐TR to discriminate between known groups and underscores its clinical sensitivity.

Regarding the discriminative capacity of the instrument, we found a higher cut‐off value (49.5) than the original recommended threshold of 35. This divergence may reflect cultural or demographic factors, as Turkish adolescents may report distress and trauma‐related symptoms more intensely on self‐report measures, resulting in higher total scores even when clinical evaluation does not warrant a PTSD diagnosis. Similarly, Hosseinnejad et al. in a systematic review of post‐earthquake populations in Iran and Pakistan, reported that studies using clinical interviews yielded lower variation and prevalence estimates of PTSD compared to self‐report methods (Hosseinnejad et al. 2022). These findings collectively suggest that cut‐off values established in the original population may not fully capture cultural and contextual differences in symptom expression. Accordingly, use of the original cut‐off score of 35 may increase the risk of overdiagnosis, particularly when diagnostic decisions rely primarily on self‐report PTSD‐RI‐5 scores. It is noteworthy that in the other Turkish validation study, Küçükardalı et al. reported very high sensitivity (96%) but comparatively lower specificity (75%) for the original cut‐off of ≥ 35 (Kucukardali et al. 2025). Thus, empirically derived cut‐offs based on Turkish data may better reflect the clinical presentation and diagnostic accuracy in this population. However, it should be emphasized that the PTSD‐RI‐5 is primarily a screening and symptom‐severity assessment tool, whereas definitive individual diagnosis should be based on a clinician‐led clinical evaluation, ideally supported by a structured or semi‐structured diagnostic interview. Further validation in larger, independent Turkish samples is needed to determine the extent of this risk and to establish the most appropriate cut‐off score for clinical use.

Our logistic regression results closely mirror the main patterns reported in earlier child/adolescent‐focused PTSD studies. First, the positive dose‐response association between overall PTSD severity and functional problems replicates Steinberg et al. (2013), who showed that each incremental rise in PTSD‐RI total score significantly increased the odds of academic and behavioral difficulties in a large clinic sample. Second, in line with findings from the National Child Traumatic Stress Network (Steinberg et al. 2019), we again observed a clear dose–response pattern: as PTSD‐RI scores rose, functional difficulties in home life, school performance, peer relations and developmental progress became progressively more likely. Even modest symptom increases therefore translated into tangible everyday impairment, underscoring the clinical value of close symptom monitoring. Bartels et al. found that Category D accounted for more than 40% of the variance in overall functional impairment (Bartels et al. 2024). Similarly, in our study, Category D predicted impairment in peer relationships and developmental progression, whereas Category E predicted impairment in home and school functioning. Unlike Bartels et al., however, Category C also emerged as a significant predictor of home impairment in our study. Finally, the absence of a significant contribution from Category B was consistent with their findings of weak or non‐significant associations between intrusion symptoms and functional impairment.

A few limitations of our study warrant discussion. First, our sample was predominantly female, limiting the generalizability of the findings to male youth. Although our sample size was adequate, it can be argued that there was insufficient diversity in certain sociodemographic and clinical characteristics, such as types of traumas and roles undertaken during the trauma. Larger, more demographically balanced samples with varied trauma exposures would be beneficial.

## 5 Conclusions

In summary, the PTSD‐RI‐5‐TR demonstrated adequate reliability and validity following adaptation, maintaining its original psychometric properties. Additional enhancements to Categories B, C, and the Dissociative Subtype scale could further bolster its reliability performance. Clinicians should interpret avoidance and intrusion symptoms with caution, as these subcategories demonstrated relatively lower reliability in this study. In Turkish clinical settings, repeated assessment and careful clinical monitoring of these symptom domains may help ensure more accurate evaluation and treatment planning. Overall, higher total PTSD scores and higher scores in the C, D, and E symptom clusters may indicate a greater likelihood of functional impairment.

## Author Contributions

**Melek Ince:** conceptualization, investigation, writing – original draft, writing – review and editing, data curation. **Fatma Sibel Durak:** supervision, project administration, writing – original draft, writing – review and editing, methodology. **Bugra Ince:** methodology, validation, formal analysis, writing – review and editing, writing – original draft, visualization. **Alan Steinberg:** conceptualization, methodology, validation, writing – original draft, writing – review and editing.

## Funding

The authors have nothing to report.

## Ethics Statement

Child and Adolescent Psychiatry Clinic at Dr. Behçet Uz Children's Hospital, University of Health Sciences, İzmir, Türkiye. Data was collected between October 2023 and August 2024. The study was approved by the local institutional ethics committee (Decision No. 2023/12‐06) and conducted in accordance with the Declaration of Helsinki.

## Consent

Written informed consent was obtained from all participants and their legal guardians.

## Conflicts of Interest

The authors declare no conflicts of interest.

## Permission to Reproduce Material From Other Sources

The authors have nothing to report.

## Data Availability

The data supporting the findings of this study are openly available in Mendeley Data at: Ince, Melek; Ince, Bugra (2025), “Turkish UCLA PTSD‐RI‐5 dataset”, Mendeley Data, V1, doi: 10.17632/7k9g64j5xz.1. All datasets have been fully anonymized to protect participant privacy, and no identifying information is included. The dataset contains item‐level responses, total and subscale scores, and demographic variables (e.g., age, gender, trauma exposure type) for participants.
